# Angular Pregnancy: A Case Report

**DOI:** 10.7759/cureus.52295

**Published:** 2024-01-15

**Authors:** Urvashi R Jainani, Rajendra Shitole, Pojala Ramyapriya, Jyotsna Patil

**Affiliations:** 1 Obstetrics and Gynaecology, Dr. D. Y. Patil Medical College, Hospital & Research Centre, Dr. D. Y. Patil Vidyapeeth, Pune, IND; 2 IVF and Endoscopy Centre, Dr. D. Y. Patil Medical College, Hospital & Research Centre, Dr. D. Y. Patil Vidyapeeth, Pune, IND

**Keywords:** uterine rupture, angular pregnancy, operative hysteroscopy, hysterectomy, ectopic pregnanacy

## Abstract

Angular pregnancy, a rare condition, marked by implantation positioned medially to the uterotubal junction within the lateral angle of the endometrial cavity poses a risk of severe complications, such as uterine rupture, placental retention, postpartum hemorrhage, and even necessitating hysterectomy, all of which can be fatal. Distinguishing angular pregnancy from other emergent conditions, particularly interstitial and cornual pregnancies, is crucial due to similar presentations and difference in embryo viability, risk, and management. While angular pregnancies can progress to term, they are associated with an elevated complication rate. Here, we present a case of primigravida with angular pregnancy who opted for evacuation under hysteroscopic guidance subsequent to unsuccessful pregnancy.

## Introduction

First described by obstetrician Howard Kelly in 1898, angular pregnancy involves ectopic implantation within the lateral angle of the endometrial cavity, medial to the uterotubal junction [1]. The embryo's position distinguishes angular from interstitial pregnancies, characterized by lateral uterine enlargement with upward and outward displacements of the round ligament in angular pregnancy [2].

The signs, symptoms, and associated complications become severe with increasing proximity of the implantation site to the lateral altitude of the uterus [3]. Angular pregnancy poses a continuous and elevated risk throughout gestation and delivery, leading to severe complications, such as chronic pelvic pain, placenta accreta spectrum (PAS), spontaneous abortion, uterine rupture, and profound bleeding that may necessitate hysterectomy [4]. Due to the inherent challenges in diagnosis, numerous cases may remain undetected, highlighting the inherent risks associated with angular pregnancies. The embryonic placement within the lateral wall endometrial thickness of the uterus in angular pregnancies results in a continuous intracavitary endometrial line surrounding the embryo [5]. While the embryo may develop within the uterus in some instances, unfavorable conditions may lead to its abortion [6]. Placental localization typically adheres to the uterine wall in the second and third trimesters, but in cases of advanced gestational age, a thickened placenta in an asymmetrically confined area of the uterine angle should raise suspicion for angular pregnancy. The growth of the placenta in these restricted areas is considered a major contributor to associated abnormalities, including a thickened placenta, asymmetric uterine appearance, PAS, local muscular weakness, and non-vertex fetal presentation [4]. The accurate identification of angular pregnancies using ultrasonography is challenging due to the inability to visualize the major anatomic marker, the round ligament. However, endovaginal ultrasonography, particularly in the early gestational weeks, is a reliable method for detecting angular pregnancies [7].

## Case presentation

A 29-year-old primigravida woman, seven weeks pregnant, presented to the Obstetrics and Gynecology Department with an ultrasound indicating an angular pregnancy accompanied by fetal bradycardia. She had a two-year married life and conceived spontaneously. Her medical, surgical, and drug history revealed no significant issues. General examination revealed stable vitals (pulse: 76 bpm, BP: 116/84 mmHg) with no signs of pallor. Cardiovascular and respiratory sounds were normal, and the abdomen was soft and non-tender. As per vaginal examination, the uterus was normal in size, retroverted, with bilateral fornices free, and non-tender, and no bleeding was present.

On admission, repeat ultrasonography revealed early intrauterine embro demise in the context of angular pregnancy (crown-rump length (CRL) 3.5 mm, corresponding to six weeks one day with absent fetal heart activity). Blood count and serologies were within normal ranges (Hb: 12.9 g/dL, WBC: 11,300/μL, platelet count: 3.67 lakhs/μL), with serology (human immunodeficiency virus (HIV), hepatitis B surface antigen (HBsAg), hepatitis C virus (HCV), and venereal disease research laboratory (VDRL)) non-reactive. Due to the unsuccessful pregnancy, the patient opted for evacuation under hysteroscopic guidance. Considering the potential for uterine perforation during hysteroscopic evaluation, a combined laparoscopic and hysteroscopic procedure was planned for termination, diagnosis, and monitoring for uterine perforation.

**Figure 1:**
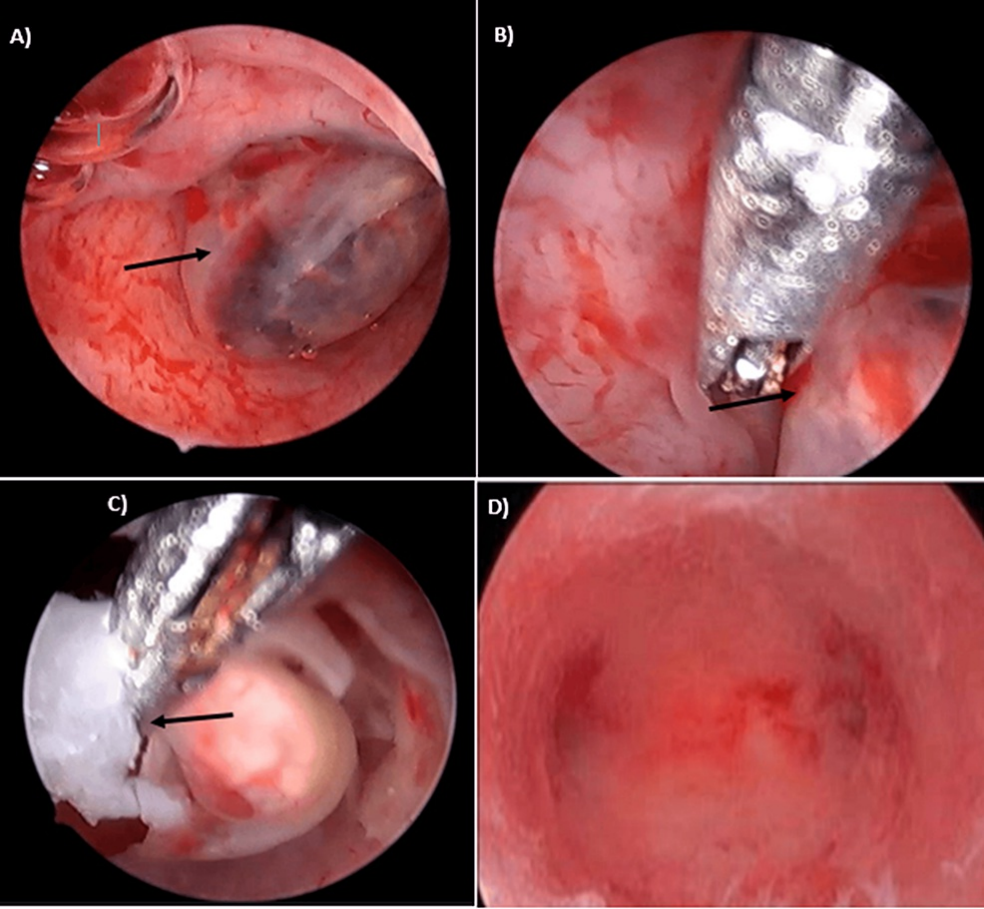
Intraoperative pictures during hysteroscopy A) Hysteroscopic picture showing the g-sac (indicated by the arrow). B) Separation of the g-sac during hysteroscopy. C) Separated g-sac being picked up. D) Empty uterine cavity confirming a successful excision of the g-sac.

During the procedure, visualization of the pouch of Douglas revealed minimal fluid. Laparoscopic visuals of the uterus were normal, with the left round ligament insertion remaining lateral to the pregnancy. No abnormal vascular changes were noted, and the extrinsic left tube appeared normal. Hysteroscopic examination identified a gestational sac (GS) over the left angle of the uterus (Figure 1A). G-sac excision was performed (Figure 1B, 1C), and the pregnancy tissue underwent histological evaluation. Subsequent hysteroscopic examination confirmed successful excision (Figure 1D), and hemostasis was achieved. The patient tolerated the procedure well, progressing to recovery for observation. Serial serum beta-HCG levels were monitored post-surgery, measuring 23,577 mIU/mL, 5864 mIU/mL, and 2751 mIU/mL on the first, second, and third days, respectively, with no procedure-related complications. The patient was discharged three days post-surgery, and serum beta-HCG confirmed negativity after one week.

## Discussion

Hysteroscopy, as a diagnostic and therapeutic tool, offers enhanced visualization of the uterine cavity, reducing intraoperative and postoperative complications and shortening hospitalization times. Specifically, angular pregnancy, located in the upper lateral side of the uterine cavity, can be effectively visualized during hysteroscopy [7].

A report by Meichen et al. (2021) highlights the utility of hysteroscopy in diagnosing and treating angular pregnancies. In two clinical cases, operative hysteroscopy was done for incomplete abortion, and the presence of angular pregnancy was successfully identified and treated with the assistance of hysteroscopy. A comprehensive review conducted by Jansen and Elliott (1981), encompassing 39 cases of suspected angular pregnancies, reported notable findings. Among the cases, 38.5% (10 of 26) resulted in spontaneous or missed abortions, and 13.6% (three of 22) were associated with uterine rupture. Recurrent bleeding throughout pregnancy was identified as a concern. Angular pregnancy was correlated with an increased risk of adverse outcomes, including preterm delivery, placental abruption, growth restriction, and postpartum endometritis. These insights emphasize the clinical significance of hysteroscopy in the context of angular pregnancies, aiding both diagnosis and therapeutic interventions [8].

Jansen and Elliot (1981) proposed evaluation criteria for angular pregnancy, encompassing painful asymmetric uterine enlargement, lateral uterine distension with or without rupture, and the retention of the placenta in the uterine angle. These criteria serve as essential benchmarks for distinguishing angular pregnancy from other conditions with similar presentations.

## Conclusions

Early pregnancy termination in cases involving angular pregnancy may be more secure. However, an inaccessible implantation site may necessitate difficult curettage. As a result, the preferred treatment methods include hysteroscopy and/or laparoscopy-guided curettage and treatment with methotrexate. The understanding of risk factors, clinical presentation, and diagnostic strategies for angular pregnancy contributes to improved patient outcomes and highlights the importance of interdisciplinary collaboration. Future research should continue to refine diagnostic approaches and therapeutic strategies, enhancing the ability to address this unique condition of ectopic pregnancy to preserve reproductive health outcomes for affected individuals.

## References

[1] Hasanzadeh M, Dadgar S, Arian Y, Yousefi Y (2017). Angular ectopic pregnancy presenting as rupture of lateral wall of the uterus: late presentation in gestation week 20. Iran J Med Sci.

[2] Densley A, Shonnard M, Conklin M, Menghani V, Reddy-Moolamalla S (2023). Angular and interstitial ectopic pregnancies: a clarification of terms and literature review. Curr Probl Diagn Radiol.

[3] Tanaka Y, Mimura K, Kanagawa T (2014). Three-dimensional sonography in the differential diagnosis of interstitial, angular, and intrauterine pregnancies in a septate uterus. J Ultrasound Med.

[4] Alanbay İ, Öztürk M, Karaşahin KE, Yenen MC (2016). Angular pregnancy. Turk J Obstet Gynecol.

[5] Arleo EK, DeFilippis EM (2014). Cornual, interstitial, and angular pregnancies: clarifying the terms and a review of the literature. Clin Imaging.

[6] Sokalska A, Rambhatla A, Dudley C, Bhagavath B (2023). Nontubal ectopic pregnancies: overview of diagnosis and treatment. Fertil Steril.

[7] Meichen Y, Jing F, Lingyun Z, Jianwei Z (2021). Two cases of angular pregnancy with incomplete abortion treated with hysteroscopy: a case report and review of literature. BMC Surg.

[8] Jansen RP, Elliott PM (1981). Angular intrauterine pregnancy. Obstet Gynecol.

